# Hormonal Regulation and Expression Profiles of Wheat Genes Involved during Phytic Acid Biosynthesis Pathway

**DOI:** 10.3390/plants4020298

**Published:** 2015-06-11

**Authors:** Sipla Aggarwal, Vishnu Shukla, Kaushal Kumar Bhati, Mandeep Kaur, Shivani Sharma, Anuradha Singh, Shrikant Mantri, Ajay Kumar Pandey

**Affiliations:** Department of Biotechnology, National Agri-Food Biotechnology Institute, C-127 Industrial Area, S.A.S-Nagar, Phase-8, Mohali, Punjab 160071, India; E-Mails: sipla@nabi.res.in (S.A.); shukla@nabi.res.in (V.S.); kaushal@nabi.res.in (K.K.B.); bedi.mandeep5@gmail.com (M.K.); shivanisharma.mtech2012@gmail.com (S.S.); anuradha@nabi.res.in (A.S.); shrikant@nabi.res.in (S.M.)

**Keywords:** phytic acid, wheat, ABA, GA_3_, gene regulation, seed development

## Abstract

Phytic acid (PA) biosynthesis pathway genes were reported from multiple crop species. PA accumulation was enhanced during grain filling and at that time, hormones like Abscisic acid (ABA) and Gibberellic acid (GA_3_) interplay to control the process of seed development. Regulation of wheat PA pathway genes has not yet been reported in seeds. In an attempt to find the clues for the regulation by hormones, the promoter region of wheat PA pathway genes was analyzed for the presence of *cis*-elements. Multiple *cis*-elements of those known to be involved for ABA, GA_3_, salicylic acid (SA), and cAMP sensing were identified in the promoters of PA pathway genes. Eight genes (*TaIMP*, *TaITPK1-4*, *TaPLC1*, *TaIPK2* and *TaIPK1*) involved in the wheat PA biosynthesis pathway were selected for the expression studies. The temporal expression response was studied in seeds treated with ABA and GA_3_ using quantitative real time PCR. Our results suggested that exogenous application of ABA induces few PA pathway genes in wheat grains. Comparison of expression profiles for PA pathway for GA_3_ and ABA suggested the antagonistic regulation of certain genes. Additionally, to reveal stress responses of wheat PA pathway genes, expression was also studied in the presence of SA and cAMP. Results suggested SA specific differential expression of few genes, whereas, overall repression of genes was observed in cAMP treated samples. This study is an effort to understand the regulation of PA biosynthesis genes in wheat.

## 1. Introduction

Gene expression is largely controlled through promoters and their contributing *cis*-acting elements that are positioned upstream of the transcriptional start site of a gene. These elements contain binding sites for transcription factors involved in the initiation and regulation of transcription. Specific regulation of the transcript in tissues and cells is of high significance, especially during the plant development. Most of the regulatory sequences of plant transcripts are located approximately 1000 base pairs (bp) upstream of the transcriptional start site [1,2].

Seed development and maturation is an important event that includes the process of desiccation and dormancy [3,4]. Maturation of seeds is also accompanied by gene expression responses and multiple biochemical events [5,6]. In wheat, three stages of gene expression patterns were observed during seed development. The first phase spans from three to seven days after anthesis (DAA) that includes the formation of multi-cellular tissue structure. The second stage starts from seven to fourteen DAA, which mainly involves grain filling. Lastly, seed maturation and desiccation that spans from 21 to 28 DAA [7]. The biosynthesis of several metabolic compounds are governed by hormones that mainly includes Abscisic acid (ABA) and Gibberellic acid (GA_3_) [8,9]. The antagonistic behaviour of ABA and GA_3_ plays a crucial role during seed physiology. ABA predominately regulates both accumulation of nutrients and desiccation during seed maturation, whereas GA_3_ activates certain enzymes required for breaking the dormancy during germination [10,11,12].

Previous findings suggested that the ABA may be a positive regulator of phytic acid (PA) synthesis in developing seeds. It has been demonstrated that ABA signal transduction is mediated by inositol-1,4,5-triphosphate (IP_3_) which, in turn, regulates seed maturation [13]. Studies have shown a positive correlation between ABA concentration and PA accumulation using seed suspension cells [14]. During the early phase of wheat seed development, 14 DAA aleurone was found to be present as distinct tissue from endosperm [15], and it has been reported previously that aleurone tissue is a major site for PA accumulation in most of the cereal grains [16]. In wheat, PA pathway genes are highly induced in aleurone at 14DAA. Thus, it could be highly informative to use 14DAA seeds to study the effects of exogenous hormones on PA pathway genes.

PA biosynthesis in plants utilize two pathways which are either lipid-dependent or lipid-independent [17]. The first committed step involves the formation of inositol3-phosphate (Ins3P) from glucose-6-phosphate by myo-inositol-3-phosphate synthase (MIPS). The subsequent steps involve a sequential and ordered phosphorylation of the remaining five positions of the inositol ring by a number of kinases [18,19]. The enzymes catalyzing these phosphorylation reactions include inositol monophosphatase (IMP), inositol tris/tetraphosphate kinase (ITPK), inositol polyphosphate kinase (IPK2) and inositol-pentakisphosphate 2-kinase (IPK1) [16,17,18]. Manipulating plant inositol phosphate kinases and the transporter of PA from various plant species is a key to generate low phytate crops (*lpa*) [18,19,20,21,22]. Additionally, IMP catalyzes the production of free inositol from dephosphorylation of Ins3P [23]. Phospholipase C (PLC) is a lipid-dependent pathway enzyme involved in the formation of IP_3_ utilizing phosphatidylinositol-4,5-bisphosphate (PIP2) as a substrate [18,23]. PLC activities have been implicated in processes as diverse as signal transduction including those in guard cells [24], against pathogen response [25], during gravitropism [26], Nod factor signaling [27,28]. The control of stomatal opening/closing by the plant hormone ABA represents one of the most thorough evaluations of PLC action in plants [29]. ITPK proteins have been identified for the intermediate stage of PA biosynthesis and have shown to be important in regulating Inositol tetrakisphosphate (IP_4_) metabolism. Multiple ITPK genes have been identified from *Arabidopsis* (*AtITPK*1-4), soybean (*GmITPK*1-4), rice (*ITP5/6K*1-6) and wheat (*TaITPK*1-4) [6,19,23,30]. Only one in barley (*HvIPK*) and maize (*ZmIP5/6K*) are reported [31,32]. IPK2 is a multifunctional kinase that phosphorylates IP_3_ at the 3rd and the 6th position. One *IPK2* gene has been characterized from rice (*OsIPK2)* and wheat (*TaIPK2*) whereas, two isoforms were identified in Arabidopsis (*AtIpk2α* and *AtIpk2β*) [6,23,33]. The Inositol pentakisphosphate kinase (IPK1) is involved in the final step in phytic acid biosynthesis. This step is common to both lipid-dependent and independent pathways. One IPK1 has been characterized from rice (*OsIPK1*), *Arabidopsis* (*AtIPK1*) and wheat (*TaIPK1*) [6,23,34]. In order to understand the regulation of PA pathway genes, especially in seeds, the effect of hormones was studied previously in rice [14]. Earlier, it has been shown that the wheat MIPS gene, which is involved in earlier steps of PA biosynthesis, is inducible by ABA, salicylic acid (SA) and other abiotic stresses [35]. No additional studies regarding the hormonal regulation of the remaining wheat PA pathway genes were reported.

SA plays an important role during biotic and abiotic stress due to its ability to generate the protective response in the plants [36]. It has been known to contribute in defense signaling during seed development [37]. Recent studies suggested the role of SA in regulation of PLC in time dependent manner upon heat stress which is positively regulated by ABA treatment [38,39]. Previously, it was shown that GA_3_ and/or auxin influence the levels of cAMP suggesting their importance during seed maturation, germination and plant development [40,41]. Although, as suggested above, multiple roles were offered by SA and cAMP in cereal crops, but reports regarding their effect on the expression of PA pathway genes were unexplored. Therefore, it would be interesting to study the temporal expression of genes involved in phytic acid biosynthesis under exogenous application of hormones (ABA or GA_3_) and biochemicals (SA or cAMP). Previously, it was shown that genes responsible for the biosynthesis of PA in wheat grains were differentially regulated [6]. However, hormonal regulation of these PA pathway genes in wheat was not reported. Thus, in the current work, we performed a comprehensive expression analysis of PA pathway genes in seeds exposed to ABA, GA_3_, SA and cAMP. Our results suggested a coordinated and time-dependent response of the phytic acid biosynthetic genes when exogenously exposed to these hormones and signaling molecules.

## 2. Results

### 2.1. *In-Silico* Analysis of the Regulatory Cis-Elements

**Figure 1:**
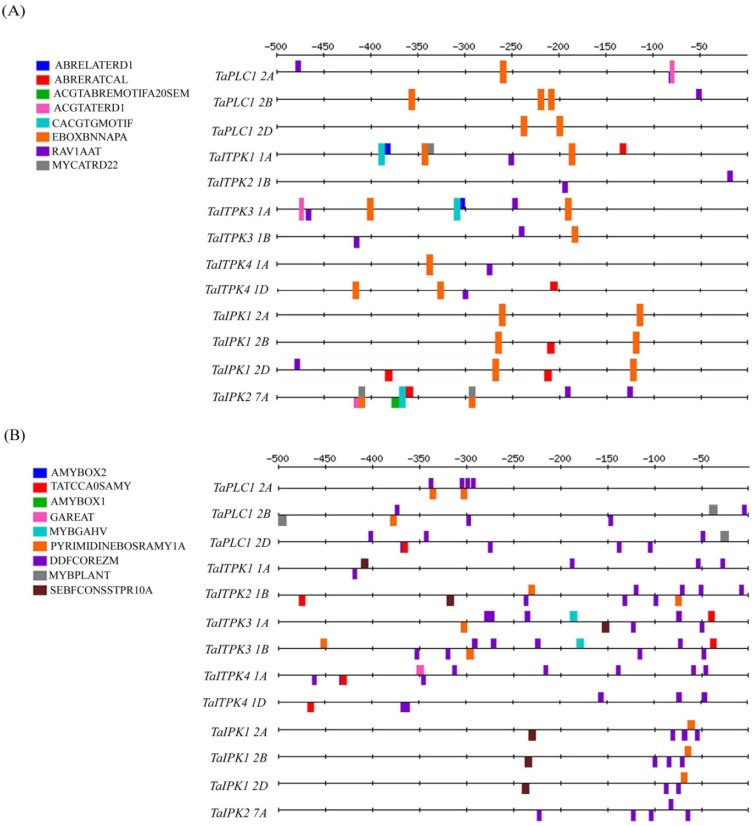

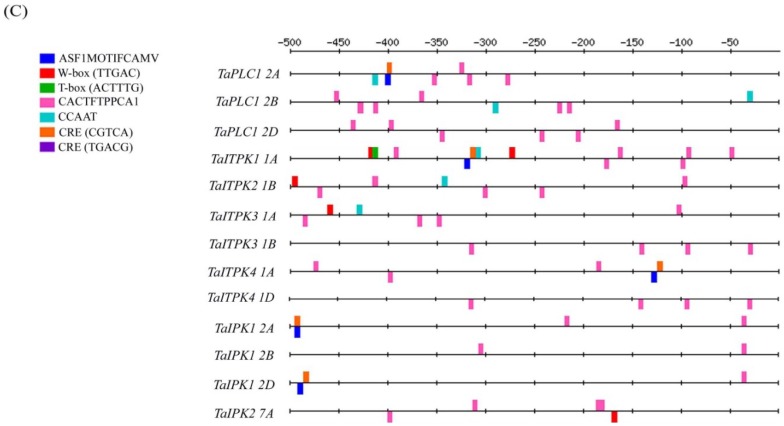
*Cis*-regulatory elements (CREs) maps in the promoter (−1 to −500) of PA biosynthesis genes. The colour indicates the position of the respective *cis*-elements. (**A**) CREs map generated for ABA response elements; (**B**) CREs map generated for GA_3_response elements; (**C**) CREs map generated for SA and cAMP response elements.

In order to elucidate the mechanism of transcriptional regulation of wheat PA pathway genes, analysis of their promoter region was performed for the *cis*-elements. A 1.0 kb sequence upstream to open the reading frame of *TaITPK1* (Ta.70767), *TaITPK2* (CA18510.1), *TaITPK3* (Ta.39455), *TaITPK4* (Ta.36061), *TaIPK1* (Ta.41955), *TaIPK2* (Ta.35113) and *TaPLC1* (Ta.55212) was identified and subjected to PLACE analysis [6,42]. *TaIMP* was not included in promoter analysis due to its inadequate information in the International Wheat Genome Sequencing Consortium (IWGSC). Promoter analysis showed the presence of multiple *cis*-elements for each of these genes [43]. For the current study, we included the *cis*-elements of the genes those were responsive to ABA, GA_3_, SA and cAMP (Table A1). In general, we observed the presence of multiple numbers of responsive elements for hormones in the PA pathway genes. For more clarity, the selected *cis*-regulatory elements (CREs) were mapped on the basis of their location in the upstream gene region (−1 to −500 bp) using Regulatory Sequence Analysis Tools (RSAT) (Figure 1).

We observed that, ABA responsive elements, EBOXBNNAPA and *RAV1AAT* were ubiquitously present in all of the PA pathway genes. For the remaining ABA responsive elements, either one or the other elements were present in the promoter of the homoeologous sequences. Out of all the PA pathway genes, *TaPLC1* and *TaIPK1* promoter elements showed highest density for the presence of ABA responsive elements. This suggests that the transcript accumulation of these genes could be easily influenced by the presence of ABA. Similarly, hormonal responsive elements for GA_3_ were also identified. Out of all the GA_3_ responsive elements, *DOFCOREZM* was prevalent in the promoter regions of the wheat PA pathway genes. The presence of high density of *DOFCOREZM* elements in all the genes suggested its importance in seeds.

In order to gather more information regarding their possible response towards signaling molecules, their promoter was also analyzed for SA and cAMP *cis*-elements. The known SA responsive elements that are primarily involved in repression *i.e.*, *CACTFTPPCA1* and *CCAAT* were present in all the genes but the highest number was observed in *TaPLC1*. This reinforces that the activity of PLC is regulated in the presence of stress generated signalling events. Promoter of all the wheat PA pathway genes also showed the presence of the cAMP response elements.

### 2.2. Hormonal Regulation of PA Pathway Genes

In order to study the temporal response of wheat PA biosynthesis pathway genes, seeds of 14 DAA were exposed to ABA and GA_3_ respectively. Subsequently, qRT-PCR was performed for eight genes involved in PA biosynthesis in wheat after treatment with respective hormones for 30 min, 45 min and 60 min post incubation. *TaMIPS* was not included in the current study since it has been characterized in detail under different abiotic stresses that include ABA, SA, NaCl and cold temperature [35]. Primers that are capable of amplifying the transcript from all the three genomes (for *TaIPK1* and *TaPLC1*) were carefully chosen unless the gene is present as a single copy (Table A2) [6,42].

**Figure 2:**
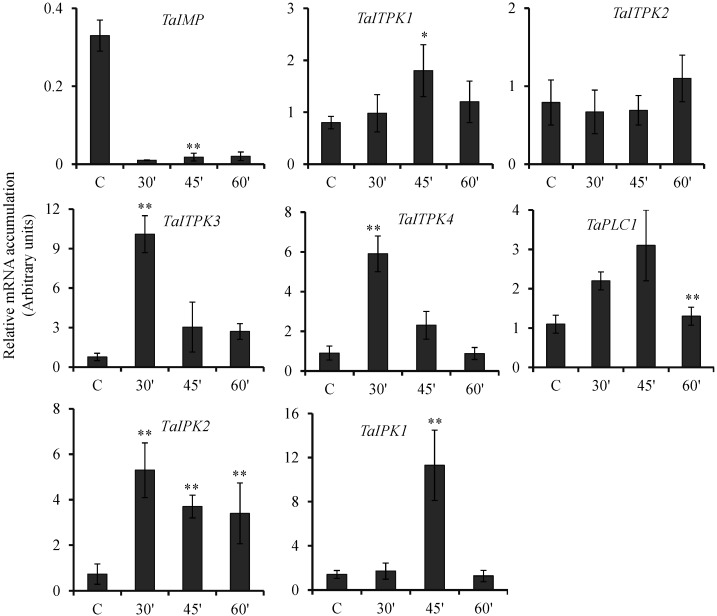
Effect of ABA on the expression of PA pathway genes. Relative fold expression of PA pathway genes in wheat grains exposed to ABA (100 μM) for 30, 45 and 60 min. After the exogenous application, the seeds were collected and RNA extraction was performed. The cDNA templates were prepared from 2 µg of DNase free RNA. Ct values obtained were normalized against *Ta18SrRNA* expression. Each bar indicates the mean of three to four replicates with the indicated standard deviation of the mean. ** indicates significant difference at *p* ≤ 0.01 and * indicates significant difference at *p* ≤ 0.05 with respect to control.

Relative fold expression levels were plotted with respect to control seeds. No significant changes in the expression of untreated samples during the time course were observed [43]. Upon exposure to ABA, wheat PA biosynthesis genes showed differential regulation during the course of the experiment (Figure 2). No significant change in the expression level of *TaITPK2* was observed. *TaITPK1*, *TaITPK3*, *TaITPK4*, *TaPLC1*, *TaIPK2* and *TaIPK1* showed significant increase in the level of transcript at different time points. Among all the genes, the highest fold induction was observed for *TaITPK3* and *TaIPK1* (~10 folds) (Figure 2). Surprisingly, the expression of *TaIMP* was repressed as compared to control seeds. Exposure to ABA resulted in induction of six out of the eight genes studied.

**Figure 3:**
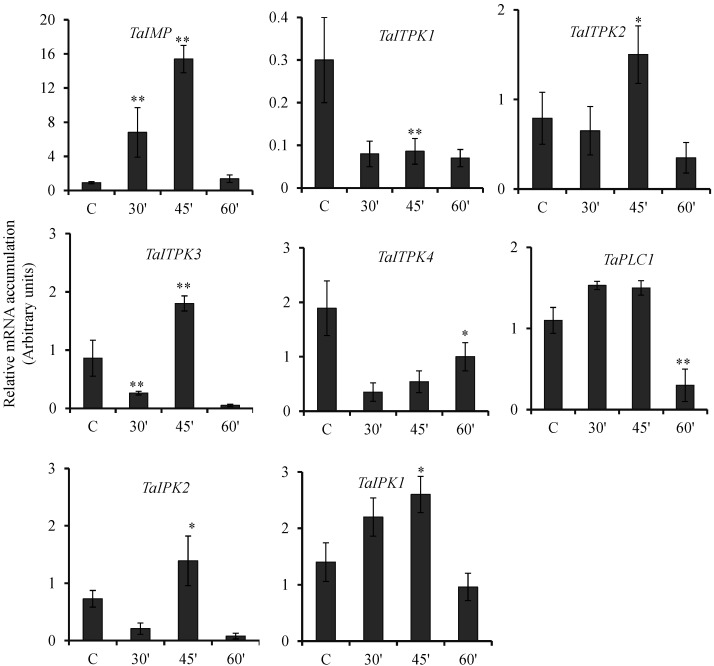
Effect of GA_3_ on the expression of PA pathway genes. Relative fold expression of PA pathway genes in wheat grains exposed to GA_3_ (60 μM) for 30, 45 and 60 min. After the exogenous application, the seeds were collected and RNA extraction was performed. The cDNA templates were prepared from 2 µg of DNase free RNA. Ct values obtained were normalized against *Ta18SrRNA* expression. Each bar indicates the mean of three to four replicates with the indicated standard deviation of the mean. ** indicates significant difference at *p* ≤ 0.01; * indicates significant difference at *p* ≤ 0.05 with respect to control.

Wheat seeds exposed to GA_3_ showed consistently decreasing expression level for *TaITPK1* and *TaITPK4* (Figure 3). *TaIMP* was induced at the early time points studied (30 min and 45 min) and subsequently showed decreases in the transcript after 60 min post incubation. For the remaining genes, *TaITPK2*, *TaITPK3*, *TaPLC1*, *TaIPK2* and *TaIPK1* showed differential expression responses. Interestingly, expression data suggested a similar pattern of expression for *TaITPK3* and *TaIPK2* although at different fold levels. Likewise, the abundance of all the wheat PA biosynthesis genes except *TaIMP* was reduced at the last time point studied after exposure to GA_3_ (Figure 3). Overall, GA_3_ showed suppression of the five genes (*TaITPK1*, *TaITPK3*, *TaITPK4*, *TaPLC1* and *TaIPK2*) that were induced by ABA, suggesting the antagonistic effects of these hormones in grains.

### 2.3. Expression Pattern of PA Pathway Genes in Presence of SA and cAMP

**Figure 4:**
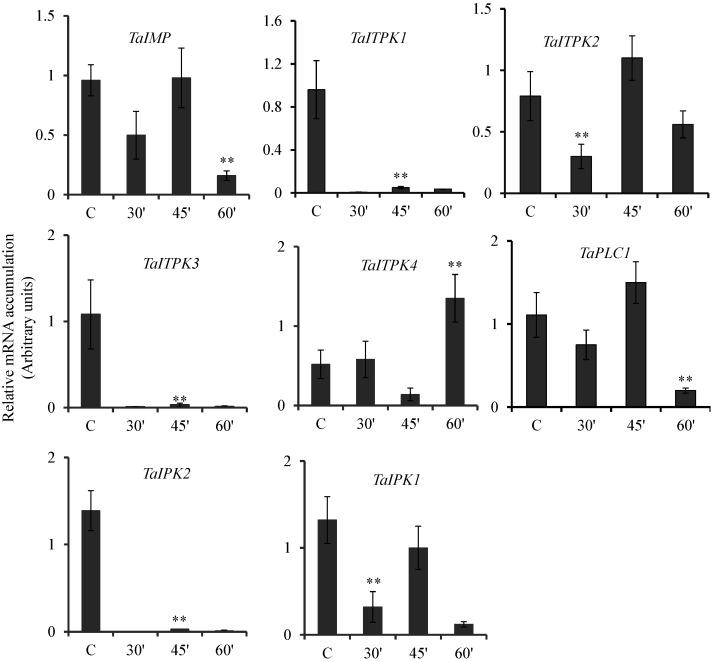
Effect of SA on the expression of PA pathway genes. The relative expression of PA pathway genes in wheat grains exposed to SA (2 mM) for 30, 45 and 60 min. After the exogenous application, the seeds were collected and RNA extraction was performed. The cDNA templates were prepared from 2 µg of DNase free RNA. Ct values obtained were normalized against *Ta18SrRNA* expression. Each bar indicates the mean of three to four replicates with the indicated standard deviation of the mean. ** indicates significant difference at *p* ≤ 0.01; * indicates significant difference at *p* ≤ 0.05 with respect to control.

Multiple studies have suggested that plant resistance responses are mediated by SA [36]. Few reports also suggested that during defense activation against pathogens, PA pathway genes, especially PLC is differentially regulated. To date, no clear link of SA with the regulation of the PA pathway has been reported. Similarly, cAMP being a major signaling molecule was also included in the current study. Temporal expression of PA pathway genes was analyzed in wheat seeds exposed to SA and cAMP. Expression profile suggested significant repression in the transcript levels of *TaIMP*, *TaITPK1*, *TaITPK3*, *TaPLC1*, *TaIPK2* and *TaIPK1* when exposed to SA as compared to their respective controls (Figure 4). Only *TaITPK4* was induced at 60 min post incubation with SA. This suggested - *TaITPK4* was induced by SA in a time dependent manner. *TaITPK2* showed significant decrease of the transcript at the initial time point (30 min), but this repression was recovered at 45 min. Subsequently, at 60 min, the expression returned to the basal level (Figure 4).

**Figure 5:**
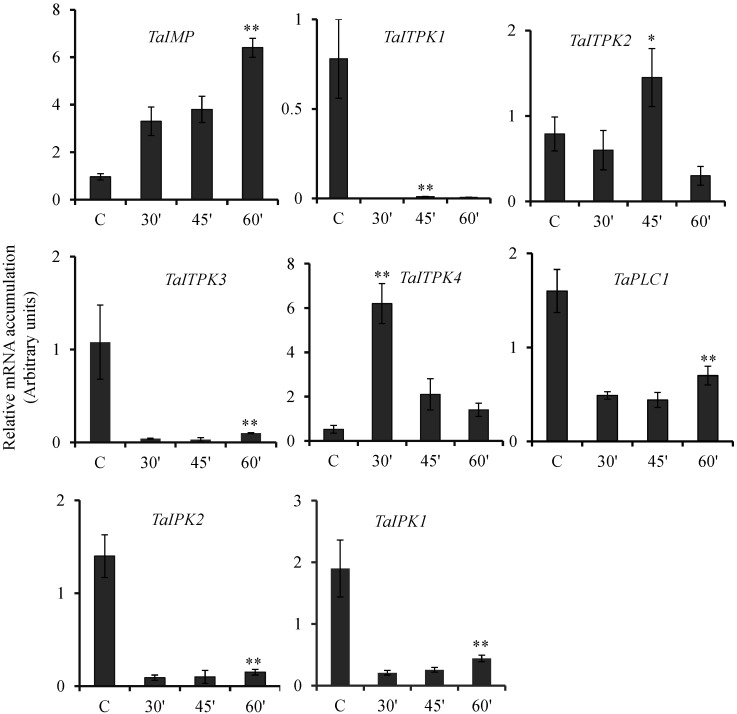
Effect of cAMP on the expression of PA pathway genes. The relative expression of PA pathway genes in wheat grains exposed to cAMP(20 μM) for 30, 45 and 60 min. After the exogenous application, the seeds were collected and RNA extraction was performed. The cDNA templates were prepared from 2 µg of DNase free RNA. *Ct* values obtained were normalized against *Ta18SrRNA* expression. Each bar indicates the mean of three to four replicates with the indicated standard deviation of the mean. ** indicates significant difference at *p* ≤ 0.01; * indicates significant difference at *p* ≤ 0.05 with respect to control.

Seeds exposed to cAMP showed a concurrent increment in the expression levels of *TaIMP* with the time points studied. At the last time point, this increase in the level of transcript corresponds to ~5 to 6 folds as compared to the control seeds (Figure 5). *TaITPK4* showed induction in their transcripts only at 30 min post incubation. Similarly, *TaITPK2* showed a slight increase in the transcript levels at 45 min, although the expression reaches the basal level at 60 min. In contrast, *TaITPK1*, *TaITPK3*, *TaPLC1*, *TaIPK2* and *TaIPK1* showed significant down regulation for the respective transcripts (Figure 5). This down regulation of PA pathway genes was similar during the time course of treatment. Overall, these data suggested that wheat PA biosynthesis genes responded differently when exposed to SA or cAMP.

### 2.4. Expression Profile Analysis of Genes during Other Biotic and Abiotic Stresses

Multiple studies have suggested the function of plant hormones as central integrators in reprogramming developmental and stress adaptive environments [44,45,46,47,48]. To study the total effect of these biotic and abiotic stresses on the wheat PA biosynthesis pathway genes, GENEVESTIGATOR database searches were performed. The corresponding probeset IDs for each gene was retrieved from Plexdb on the basis of alignment and sequence identity (Table A3).

**Figure 6:**
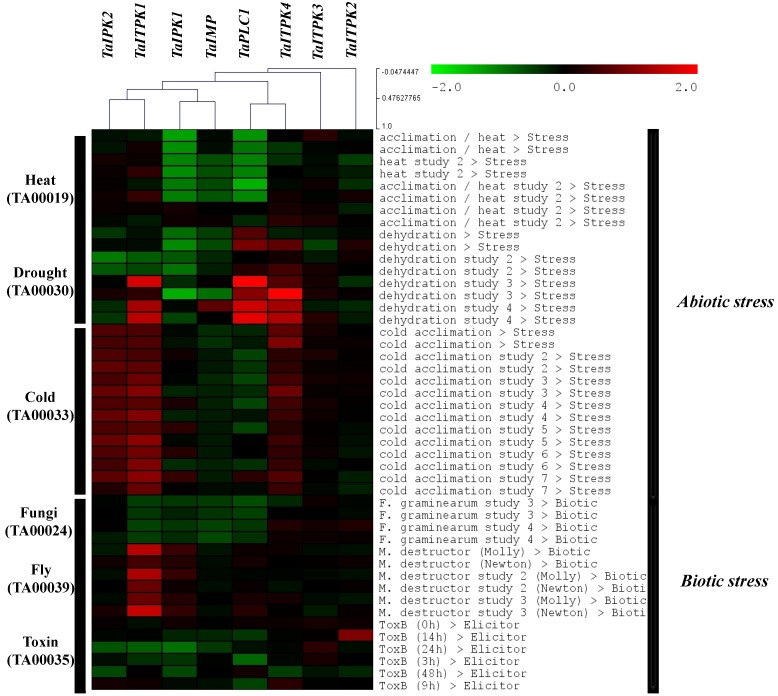
Microarray based expression profiles of selected PA pathway genes. Foliar expression of seven probe sets was analyzed against 250 samples representing heat, drought, cold and biotic stress conditions. The heat map was generated using meta-analysis tool at GENEVESTIGATOR (https://www.genevestigator.com/gv/plant.jsp). Colours represent the fold change for expression.

The heat map produced after an analysis revealed differential expression pattern of genes in abiotic stress and biotic stress conditions (Figure 6). Under abiotic stress conditions, *TaITPK1* and *TaITPK4* were preferentially induced by both cold stress and drought stress. *TaIPK2* was significantly induced by cold stress, whereas *TaIMP* and *TaPLC1* were generally downregulated in cold and heat stress environments. *TaPLC1* was significantly up-regulated under drought stress. *TaIPK1* was found to be down-regulated under heat stress study. To correlate the expression pattern of PA pathway genes under biotic stress conditions, studies on *Fusarium graminearun* (fungi), *Mayetiola destructor* (fly) and a fungal toxin ToxB (elicitor) were included as other biotic stress studies showed no differential expression of the PA pathway genes. The heat map showed significant up-regulation of *TaITPK1* when exposed to *M. destructor*. Moreover, the rest of the genes showed slight downregulation under biotic stress conditions. Also, *TaITPK3* showed no expression change under both abiotic and biotic stress studies. This analysis suggested that beside the prime role of PA pathway genes in seeds, they also show differential response towards multiple stresses.

## 3. Discussion

Lowering the phytate content in the seeds is considered to be an important trait. Multiple reports describing the genes involved in the plant PA biosynthesis along with their functional importance have been described. Understanding the regulation of these genes remained largely unexplored. During the onset of grain maturation, the seed hormone level contributes towards the development and regulation of gene transcript [49]. Since the rate of accumulation of PA biosynthesis is higher in the early stages of seed maturation [6], we expect the effect of endogenous hormones on the expression response of PA biosynthesis genes in seeds. Thus, in the current study, we tested the effect of exogenously applied hormones (ABA and GA_3_) and signalling molecules (SA and cAMP) on the temporal regulation of the PA pathway genes. Initially, we identified the regulatory elements in the promoter of genes involved in the PA biosynthesis in wheat. The analysis supported our rationale as we observed multiple *cis*-elements in the promoter region that could be responsible for perception of either ABA, GA_3_, SA or cAMP (Table A1).

**Figure A1:**
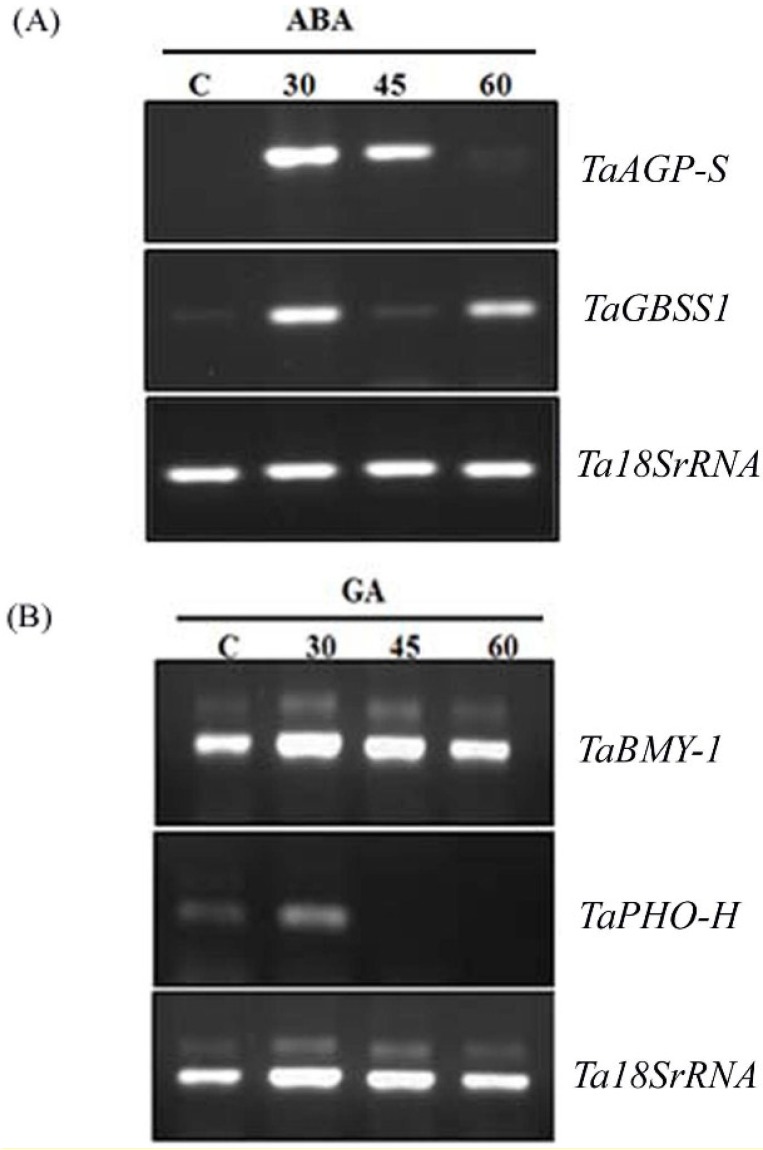
Semi-quantitative RT-PCR of wheat genes as a marker for ABA and GA_3_ treatment on seeds. (**A**) PCR amplification of *TaAGP-S* (ADP-glucose pyrophosphorylase, smaller subunit) and *TaGBSS1* (Granule-bound starch synthase 1) from 14 DAA seeds treated with ABA; (**B**) PCR amplification of *TaBYM1* (β-amylase) and *TaPHO-H* (starch phosphorylase H) in wheat seeds (14 DAA) when treated with GA_3_. After respective treatments the seeds were collected at 30 min, 45 min and 60 min post incubation. 2 µg of total RNA (DNase treated)was extracted and cDNA templates were prepared. Low-cycle semi-quantitative RT-PCR was performed along with *Ta*18SrRNA as an internal control. The amplicons were resolved on 1.2% agarose gel. The experiments were repeated twice from different biological replicates.

Our experimental setup (for ABA and GA_3_) was validated by amplifying the marker genes that included wheat amylase genes and starch synthesizing transcripts. A positive co-relation to the induction of the representative genes was observed in the respective treatment (Figure A1). Multiple copies of ABA-responsive element (RAV1AAT) and GA-responsive element (DOFCOREZM) were present in the promoters of wheat PA pathway genes suggesting their role in different physiological responses. Studies have shown that the phytic acid level is rapidly enhanced in guard cells in response to ABA [50]. Similarly, tissue specific role of phosphatidic acid signalling was also tested in barley aleurone cells [51]. These observations suggested the important role of PA pathway genes in increments of phytic acid upon exogenous ABA treatment. Enhanced PA accumulation was reported in rice suspension cells when exposed to ABA [14]. We speculate that upon ABA treatment PA accumulation could be due to the reduced breakdown of PA or its altered transport. This warrants detailed investigation for the measurement of PA in seeds treated with ABA. Interestingly, some of these *cis*-elements are consistently present at the same location within the promoters of the homoeologous gene. For example, in case of *TaIPK1*, most of the *cis*-elements in the promoters of different genomes were located at the similar positions.

One of the interesting observation was the earliest expression of *TaITPK3* and *TaITPK4* (at 30 min) followed by *TaIPK1* (at 45 min) suggesting the sequential expression cascades for the PA pathway genes. In fact, one of the intermediate pathway genes in *Arabidopsis*, *AtITPK2* was also highly induced upon ABA treatment in seeds [52]. ABA is also interlinked with the drought tolerance, for example, *OsITPK*genes also showed differential response against drought stress and ABA signaling [53]. In ABA treated wheat seeds, the expression of *TaIPK2* was consistently up-regulated at all time points which are in contrast to the expression of *AtIPK2β* that decreased upon ABA treatment [54]. Expression pattern of *OsIPK1* [14], and our results regarding *TaIPK1* suggested higher fold accumulation of their transcripts when treated with ABA. Except for *TaITPK2* and *TaIMP* all the wheat genes involved in PA biosynthesis were induced by ABA (Figure 2). It will be interesting to scan the promoter region of *TaIMP* for the ABA-responsive *cis* element. This may imply regulation of the inositol signaling pathway by ABA in developing wheat seeds is pre-dominantly active downstream of *TaIMP* product. Treatment of wheat seeds with GA_3_ resulted in down-regulation of *TaITPK1*, *TaITPK4* and *TaPLC1* (Figure 3). For *TaITPK3* and *TaIPK2*, the repression in the transcript levels was time specific. Altogether, this reinforces the observation that some of the intermediated PA pathway genes, *TaITPK1*, *TaITPK4* and *TaPLC1* are antagonistically regulated by the presence of ABA and GA_3_. Similarly, *TaIPK1* was only induced by ABA at 45 min, whereas downregulation of the gene was observed in the presence of GA_3_ at 60 min. However, the expression pattern of *TaIMP* suggests its preferential regulation by GA_3_ when compared to ABA during seed development. Previously, it has been shown that GA_3_ leads to a rapid and transient IP_3_ accumulation in barley aleurone [55] and in rice seeds, ABA alone does not alter the levels of IP_3_ [56]. This raises a question of to what extent ABA-GA_3_ antagonism regulates inositol signaling leading to phytic acid. Interestingly, recent characterization of *AtIMPL1* suggested it as a bi-functional enzyme and functionally different from *AtIMPL2* [57,58]. *AtIMPL2* does not function in the inositol signaling pathway, this functional diversification between closely related homologous enzymes warrants detailed characterization of *TaIMP* and its role in the inositol signaling pathway.

Overall, our studies indicated that some of the PA pathway genes are highly induced by ABA when compared to GA_3_. These observations are in accordance with the previous observations reported from rice suspension cells where overall induction of PA biosynthesis genes was observed in presence of ABA [14]. Altogether, our observations also support that, ABA and GA_3_ act antagonistically in developing wheat grains. One of the surprising observations made was the downregulation of *TaIMP* when exposed to ABA and SA. In contrast, two IMP in rice showed very low or no changes in their expression patterns when treated with ABA [14]. Interestingly, IMP in *Cicer arietinum* was induced when treated with ABA [59]. Our GENEVESTIGATOR data suggested that, under drought conditions, no significant changes in the expression *TaIMP* were observed. ABA has been linked to the drought conditions; thus, we anticipate that other wheat IMP may be regulated by ABA. Multiple studies have indicated the role of SA during thermo tolerance in crop plants [60]. Our studies indicated general repression of all the wheat PA pathway genes (Figure 4) in presence of SA. These observations were also reinforced by the presence of maximum number of *cis*-elements (mesophyll expression module1, CACTFTPPCA1 and heat shock element, CCAAT) that contributed for SA mediated repression (Table A1). These elements were over-represented in SA repressed genes [36]. On the similar lines, overall repression was observed for PA wheat genes when treated with cAMP (Figure 5). cAMP is an important signaling molecule [61], and their role in seed biology is still not explored to an extent. To get an insight of cAMP-mediated repression of PA pathway-related genes, further detailed studies need to be performed. Future studies will be directed to understanding the signaling steps that control the specific expression of PA pathway genes.

Analysis was done to correlate our expression studies to that of abiotic and biotic stresses (Figure 6). *TaITPK1*, *TaITPK4* and *TaPLC1* were induced not only by the ABA but also under drought conditions. This suggested that the response of these genes under drought conditions could be because of the ABA. Our hypothesis is supported by the *in-silico* observations that showed the presence of four ABA/dehydration responsive *cis-*elements (ACGTATERD1, ABRELATERD1, EBOXBNNAPA, MYCATRD22) [62]. Reduction of phytic acid in plants is generally accompanied with the enhanced susceptibility of the plant towards pathogens [63]. The differential response in the expression profiles of some genes (*TaPLC1*, *TaIPK1* and *TaITPK1*) under biotic stresses also suggested their possible role in plant defense and in other tested conditions. Altogether, these differential response profiles for exogenous hormones, signalling molecules and other stresses suggest the importance of PA biosynthesis genes in seeds and physiology of the plants.

## 4. Experimental Section

### 4.1. Plant Materials and Growth Conditions

Bread wheat cv. C306, a good processing quality Indian variety, was used for this study. For tissue sample collection, plants were grown in growth chambers under a 12 h photoperiod at 400 μmol·m^−2^·s^−1^, 70% relative humidity and 25 °C/18 °C (day/night). To study hormonal regulation, the main individual spikes of the biological replicates were tagged at first day after anthesis (DAA). The tagged spikes were harvested at 14 DAA and were used to collect seeds.

### 4.2. Exogenous Treatment of Seeds with Hormones

To study the effect of hormones, abscisic acid (ABA) and gibberellic acid (GA_3_) were used as suggested earlier with minor modifications [51,64,65]. Six to seven spikes of 14 DAA stage were used to collect seeds and were subjected to incubation with either of the treatments that include ABA (100 μM), GA_3_ (60 μM), SA(2 mM) and cAMP (20 μM) at different time intervals *i.e.*, 30, 45 and 60 min. Control seeds imbibed in CaCl_2_ were used. Three replicates containing 22–25 seeds per plate, with their respective hormonal treatments, were used for the experiment. Seeds were collected after the given time points and rapidly chilled before storage at −80 °C.

### 4.3. RNA Isolation from Plant Materials and Quantitative Real Time PCR (qRT-PCR)

RNA was extracted from wheat seeds (treated and untreated) using RNeasy Plant MiniKit (Qiagen, Valencia, CA, USA), following manufacturer instructions. Genomic DNA contamination was removed using Trubo DNA-free^TM^ kit(Ambion/Life Technologies, Grand Island, NY, USA). Transcriptor First Strand cDNA Synthesis Kit RT-PCR (Roche, Indianapolis, IN,USA) was used for cDNA preparation from two micrograms of RNA. Reverse transcription was performed using random hexamer primers following the manufacturer’s guidelines. Primers capable of amplying all the homeolog of the given gene were used for the study (Table A2). Thus, in the current study, an additive effect of the transcripts arising from all the different genome was represented unless the gene is present as a single copy.

Quanti-Tect SYBR Green RT-PCR Master mix (Qiagen, Valencia, CA, , USA) was used in qRT-PCR reactions,upto 45 cycles on 7500 Fast & 7500 Real-Time PCR System (Applied Biosystems, Foster City, CA, USA). Five replicates from each biological sample were used to perform qRT-PCR analysis. For PCR reaction, four replicates for each gene were amplified from two independent cDNA preparations. Relative expression level was quantified using 2^−ΔΔCt^ method after normalizing Ct values against 18 s rRNA expression [66,67].

### 4.4. *In-Silico* Analysis of Cis-Element Search in Upstream Region of Wheat Genes

The full length cDNA sequence of respective genes was confirmed using NCBI (Blastx). The promoter sequences were obtained by comparison with wheat genomic sequences. One-thousand bp upstream of 5'-terminus of each cDNA were selected by using genomic data from IWGSC (http://wheat-urgi.versailles.inra.fr/Seq-Repository/). Due to limited genomic sequence information about wheat genome, promoter sequences for *TaITPK3*, *TaITPK4* and *TaIPK2* were derived by using *Triticum Urartu* (A-genome) or *Aegilops tauschii* (D-genome) genomic database from NCBI or IWGSC. Table A4 describes the sequence IDs for each gene that were used to extract promoter regions. Promoter sequence for *TaIMP* could not be retrieved due to inadequate information in IWGSC. All possible *cis*-elements known in plants were searched by using PLACE database (http://www.dna.affrc.go.jp/PLACE/). The *cis*-regulatory elements (CREs) were mapped −1 to −500 bp upstream gene region using RSAT tool (http://www.rsat.eu/).

### 4.5. GENEVESTIGATOR Analysis of PA Pathway Genes

The spatial distribution of genes involved in phytic acid synthesis was studied using seven probe set IDs (Table A3). The expression pattern of PA pathway genes was analyzed across 250 samples representing heat, drought, cold and biotic stress conditions. Microarray expression IDs from PlexDB (TA00019, TA00030, TA00033, TA00024, TA00039 and TA00035) representing the above stresses was used for generating heat maps. They were clustered into heat maps to compare expression of candidate genes using GENEVESTIGATOR software [68]. In addition, Euclidean distance matrix algorithms using optimal leaf-ordering was used to hierarchically cluster the expression data.

## 5. Conclusions

In conclusion, we have observed that ABA primarily controls the expression of wheat PA pathway genes. These genes are also regulated under multiple stresses and by other signalling molecules. Such hormone-based regulation studies of multiple genes involved in biological pathways could be important for genetic interventions aimed at trait development. Additionally, our expression profiles for the wheat PA pathway genes suggested the antagonistic relation of ABA and GA_3_ in seeds.

## Appendix

**Table A1:** Specitication of *cis*-responsive elements of ABA, GA3, SA and cAMP in genes involved during PA biosynthesis in wheat. The number below represents the occourance of the response elements in the promoter.

*cis* elements	*TaPLC1*			*TaITPK1*	*TaITPK2*	*TaITPK3*		*TaITPK4*		*TaIPK2*	*TaIPK1*		
	*2A*	*2B*	*2D*	*1A*	*1B*	*1A**	*1B*	*1A**	*1D**	*7A**	*2A*	*2B*	*2D*
**ABA RESPONSE ELEMENTS**													
*ABRELATERD1*	1	0	1	3	0	3	0	1	1	4	1	2	1
*ABRERATCAL*	1	0	1	2	1	1	0	0	1	2	2	4	4
*ACGTABREMOTIFA2OSEM*	0	0	1	0	0	0	0	0	0	1	0	0	0
*ACGTATERD1*	4	4	4	6	0	6	0	2	4	8	2	4	2
*CACGTGMOTIF*	0	0	0	2	0	2	0	0	0	2	0	0	0
*EBOXBNNAPA*	6	10	8	8	14	10	10	8	12	6	10	10	12
*RAV1AAT*	5	8	4	2	3	3	3	2	2	4	2	1	2
*MYCATRD22*	0	0	1	1	1	0	0	1	0	2	1	1	1
**GA RESPONSE ELEMENTS**														
*AMYBOX2*	0	0	1	1	0	0	0	1	0	0	0	0	0
*TATCCAOSAMY*	0	0	1	1	1	2	2	1	1	0	0	0	0
*AMYBOX1*	0	1	0	1	0	1	1	0	1	0	1	0	2
*GAREAT*	0	1	0	1	0	1	1	1	1	0	1	0	2
*MYBGAHV*	0	1	0	1	0	1	1	0	1	0	1	0	2
*PYRIMIDINEBOXOSRAMY1A*	3	1	0	0	2	1	2	0	0	0	2	3	2
*DOFCOREZM*	9	7	9	6	10	17	12	13	17	7	7	9	9
*MYBPLANT*	0	3	1	0	0	0	0	0	0	1	1	1	1
*SEBFCONSSTPR10A*	0	1	0	1	1	1	1	0	0	2	3	2	2
**SA RESPONSE ELEMENTS**													
*ASF1MOTIFCAMV*	4	1	0	1	1	0	1	4	2	2	1	0	2
*W-box (TTGAC)*	0	1	1	2	1	2	1	0	1	5	0	1	1
*T-box (ACTTTG)*	0	0	0	1	0	0	0	1	0	1	1	0	0
*CACTFTPPCA1*	10	12	15	14	12	12	11	13	10	10	8	10	8
*CCAAT*	2	2	2	4	1	1	1	1	2	1	0	0	0
**cAMP RESPONSE ELEMENTS^#^**													
*CRE (CGTCA)*	2	1	0	1	1	0	1	2	1	2	1	0	1
*CRE (TGACG)*	2	0	0	0	0	0	0	2	1	0	0	0	1

* Promoter gene sequence derived from *Triticum urartu (*A-genome*)* or *Aegilops tauschii (D-genome)*; ^#^ CRE elements consensus sequences from were manually searched in the promoter sequences [69].

**Table A2:** Primer sequences used in the current study.

Name of Gene	Primers (5'–3')	Amplicon size (in bp)
*TaITPK1*	Forward: ATGGTGGCCGAGCACCAGTCCTC Reverse: GACGACGGGCACGGAGGGGTGG	261
*TaITPK2*	Forward: AGGAGTTCGTCAACCATGGCGGCGT Reverse: GCCGCCCGCGATCTGGTTGATGAAT	247
*TaITPK3*	Forward: ACGCCATCGACCGCCTCCACAACC Reverse: CGGCGACGAGGGGCTTGGCGATGA	222
*TaITPK4*	Forward: CAGCCTGCCGGACGTGCCGAC Reverse: ATGTCGAAGTTGAACAGCTGCA	210
*TaPLCl*	Forward: CGTGCTCCTATCAACAAAGCC Reverse : CTGTTCGTCCTCATCGTCGT	317
*TaITPK2*	Forward: GCCCTACGTCACCAAGTGCCTCGC Reverse: AACCACGCCTTGAGCTCGCGCAGC	283
*TaITPK1*	Forward: ACTGGGTCTACAAGGGAGAGGGC Reverse : ACACGAACCCCGCCATCAACATGA	279
*TaLMP*	Forward: GGACCACAAGTTCATCGGCGAGG Reverse: CTCCAACGGTGGGAATCTTCCC	176
*Ta18SrRNA*	Forward: GTGACGGGTGACGGAGAATT Reverse : GACACTAATGCGCCCGGTAT	150
*TaAGP- S*	Forward: GCAAGATACACCATTCAGTAGTTGGAC Reverse : GACTGTTCCACTAGGGAGTAAAGCATC	326
*TaGBSS-I*	Forward: CTCGCCGCCAACTACGACGTC Reverse : TGCTCGGGAACTTCTCCTCCAC	268
*TaBMY*	Forward: CTAGCCAACTATGTCCAAGTCTACGT Reverse : ACTGTGGGATGGGGATGTTGACGA	370
*TaSP-H*	Forward: TGGCCAGCAAAAAGCGCCTAGC Reverse : TCTGCCTGTCTGCTGCGCTCA	183

**Table A3:** Alignment table for gene *vs*. probesets. PA pathway gene sequences were queried by Blastn against Plexdb wheat microarray database.

Query gene	Target Probe set ID	e-value	Identity (%)	Query length	Query range	Target length	Target range	Target description
*TaITPK1*	Ta.27561.1.S1_at	0.0	97.2	1038	25..938	1219	120..1036	gb|CK215630
*TaITPK2*	TaAffx.113052.1.S1_at	9e-52	86.2	876	533..770	549	139..377	gb|CA618510
*TaITPK3*	TaAffx.19998.1.A1_at	0.0	97.5	1057	490..1057	1696	616..1184	gb|CD867474
*TaITPK3*	TaAffx.19998.1.A1_at	1e-151	97.0	1057	134..431	1696	1243..1540	gb|CD867474
*TaITPK3*	TaAffx.19998.1.A1_at	1e-29	100.0	1057	49..114	1696	1560..1625	gb|CD867474
*TaITPK4*	TaAffx.121326.1.S1_at	0.0	99.0	1018	6..606	722	64..667	gb|CA690518
*TaITPK4*	Ta.27561.1.S1_x_at	3e-58	84.4	1018	54..342	1219	134..422	gb|CK215630
*TaPLC1*	Ta.13185.2.A1_at	1e-46	86.5	1876	1541..1750	224	1..204	gb|BJ214989
*TaIPK2*	Ta.30464.1.A1_at	1e-133	100.0	899	1-240	858	291-530	gb|CK214494
*TaIPK2*	Ta.30464.1.A1_at	1e-117	100.0	899	306-517	858	14-225	gb|CK214494
*TaIPK1*	TaAffx.123037.2.S1_at	0.0	96.4	1125	28-1125	1502	198-1297	gb|CA613702
*TaIMP*	Ta.14044.1.S1_at	0.0	97.5	983	1..893	1085	90..982	gb|BT009424

**Table A4:** Sequence IDs for each gene those were used to extract promoter regions.

Query gene	IWGSC ID	NCBI ID
*TaITPK1-2A*	IWGSC_1AL_v2|IWGSC_chr1AL_v2_ab_k71_contigs_longerthan_200_3977071	
*TaITPK21B*	IWGSC_1BL|IWGSC_chr1BL_ab_k71_contigs_longerthan_200_3850458	
*TaITPK3-1A**	*-*	gb|KD008473.1
*TaITPK3-1B*	IWGSC_1BL|IWGSC_chr1BL_ab_k71_contigs_longerthan_200_5693968	
*TaITPK4-1A**	*-*	gb|KD262384.1
*TaITPK4-1D**	*-*	gb|KD502010.1
*TaPLC1-2A*	IWGSC_2AL|IWGSC_chr2AL_ab_k71_contigs_longerthan_200_6422019	
*TaPLC1-2B*	IWGSC_2BL|IWGSC_chr2BL_ab_k71_contigs_longerthan_200_7936983	
*TaPLC1-2D*	IWGSC_2DL|IWGSC_chr2DL_ab_k71_contigs_longerthan_200_9907826	
*TaIPK2-7A**	*-*	gb|KD115052.1
*TaIPK1-2A*	IWGSC_2AL|IWGSC_chr2AL_ab_k71_contigs_longerthan_200_6422019	
*TaIPK1-2B*	IWGSC_2BL|IWGSC_chr2BL_ab_k71_contigs_longerthan_200_7936983	
*TaIPK1-2D*	IWGSC_2DL|IWGSC_chr2DL_ab_k71_contigs_longerthan_200_9907826	
